# Carboxyl-Functionalized Carbon Nanotubes Loaded with Cisplatin Promote the Inhibition of PI3K/Akt Pathway and Suppress the Migration of Breast Cancer Cells

**DOI:** 10.3390/pharmaceutics14020469

**Published:** 2022-02-21

**Authors:** Madalina Andreea Badea, Mihaela Balas, Mariana Prodana, Florentina Gina Cojocaru, Daniela Ionita, Anca Dinischiotu

**Affiliations:** 1 Department of Biochemistry and Molecular Biology, Faculty of Biology, University of Bucharest, 91-95 Splaiul Independentei, R-050095 Bucharest, Romania; madalina.andreea.badea@drd.unibuc.ro (M.A.B.); anca.dinischiotu@bio.unibuc.ro (A.D.); 2 Department of General Chemistry, Faculty of Applied Chemistry and Materials Science, Politehnica University of Bucharest, 313 Splaiul Independentei, R-060042 Bucharest, Romania; mariana.prodana@upb.ro (M.P.); daniela.ionita@upb.ro (D.I.); 3 Department of Anatomy, Physiology and Biophysics, Faculty of Biology, University of Bucharest, 91-95 Splaiul Independentei, R-050095 Bucharest, Romania; florentina.cojocaru@unibuc.ro

**Keywords:** triple-negative breast cancer, single-walled carbon nanotubes, phosphatidylinositol 3-kinase, protein kinase B, migration

## Abstract

PI3K/Akt signaling is one of the most frequently dysregulated pathways in cancer, including triple-negative breast cancer. With considerable roles in tumor growth and proliferation, this pathway is studied as one of the main targets in controlling the therapies’ efficiency. Nowadays, the development of nanoparticle–drug conjugates attracts a great deal of attention due to the advantages they provide in cancer treatment. Hence, the main purpose of this study was to design a nanoconjugate based on single-walled carbon nanotubes functionalized with carboxyl groups (SWCNT-COOH) and cisplatin (CDDP) and to explore the potential of inhibiting the PI3K/Akt signaling pathway. MDA-MB-231 cells were exposed to various doses (0.01–2 µg/mL SWCNT-COOH and 0.00632–1.26 µg/mL CDDP) of SWCNT-COOH-CDDP and free components for 24 and 48 h. In vitro biological tests revealed that SWCNT-COOH-CDDP had a high cytotoxic effect, as shown by a time-dependent decrease in cell viability and the presence of a significant number of dead cells in MDA-MB-231 cultures at higher doses. Moreover, the nanoconjugates induced the downregulation of PI3K/Akt signaling, as revealed by the decreased expression of PI3K and p-Akt in parallel with PTEN activation, the promotion of Akt protein degradation, and inhibition of tumor cell migration.

## 1. Introduction

Triple-negative breast cancer (TNBC) represents a special molecular subtype of breast cancer (BC) defined by the lack of three molecular biomarkers: estrogen receptor (ER), progesterone receptor (PR), and human epidermal growth factor receptor-2 (HER2) [1]. TNBC accounts for approximately 15–20% of total BC cases worldwide [1,2], is the most aggressive and heterogeneous subtype of BC [3], and, compared with other molecular subtypes of BC, it is associated with a poor prognosis, aggressive clinical behavior, low survival rates after recurrence, and early metastasis [2,4]. Moreover, TNBC tumors present high invasivity, increased expression of proliferation markers, high growth rate, and can rapidly metastasize to the brain, lungs, and liver [1,2].

One signaling pathway frequently dysregulated in TNBC and associated with uncontrolled cell proliferation, metabolism, and survival, which leads to the development and progression of cancer, is PI3K/Akt/mTOR [5]. In normal conditions, the PI3K/Akt/mTOR pathway is activated by the binding of various stimuli (growth factors, hormones, cytokines) to receptor tyrosine kinases or G protein-coupled receptors which determine the recruitment of PI3K protein to the cellular membrane. Once activated, PI3K catalyzes the phosphorylation of phosphatidylinositol 4,5-bisphosphate (PIP2) to phosphatidylinositol 3,4,5-trisphosphate (PIP3) [6,7]. Furthermore, PIP3 can activate a variety of signaling proteins through the interaction between this phosphoinositide and their PH (pleckstrin homology) domain. The most relevant downstream effector of PI3K is the serine/threonine kinase Akt, also known as protein kinase B. This protein causes the activation of the mTORC1 complex and induces protein synthesis, cell growth, proliferation, and survival [7,8,9]. Moreover, the PI3K/Akt/mTOR pathway is negatively regulated by phosphatases. An important tumor suppressor of this pathway is represented by the phosphatase and tensin homolog (PTEN) which induces the dephosphorylation of PIP3 to PIP2, interrupting further signaling and resulting in cell growth inhibition [10]. The TNBC is characterized by hyper-activation of the PI3K/Akt/mTOR pathway, which is correlated with the loss or inactivation of PTEN, PI3K mutation or amplification, and Akt and/or mTOR mutation [8].

The negative expression of ER, PR, and HER2 receptors renders it impossibile to use endocrine and targeted anti-HER2 therapies in the treatment of TNBC and reduces significantly the range of therapeutic possibilities. Chemotherapy remains the main systemic treatment option in TNBC [1,3], along with anthracyclines, taxanes, and platinum-based chemotherapeutics [11,12]. 

In the treatment of TNBC, two platinum-based drugs are currently used: cisplatin and carboplatin, which are administrated by intravenous injection. Cisplatin (CDDP) is given intravenously as a short-term infusion of normal salinity for the treatment of solid and hematological malignancies, and its cytotoxic action is induced by interference with DNA replication, causing the death of cancerous cells [13,14]. However, chemotherapy presents the disadvantages of poor efficacy, side effects, high recurrence, and low survival rate, which underlines the necessity of developing novel therapeutic strategies [1,3]. Over the years, various TNBC treatment approaches were tested—targeted therapy (using inhibitors of poly ADP ribose polymerase (PARP), phosphatidylinositol 3-kinase/protein kinase B (PI3K/Akt), mitogen-activated protein kinases (MAPK), cyclin-dependent kinase CDK), immunotherapy, along with the development of antibody–drug conjugates—but no significant improvement was observed [3,15,16]. Therefore, the development of more specific and targeted therapies for TNBC is a clinical necessity, a promising tool in this direction being represented by nanomedicine and the use of nanoparticles (NPs) as vehicles for various drugs. 

Various types of NPs have been studied as delivery systems for medical agents in the treatment of BC: quantum dots [17], iron oxide NPs [18], liposomes, dendrimers, polymeric micelles or carbon NPs [19]. The use of NPs in cancer therapy improved the delivery of drugs to a specific target tumor site, with a higher efficiency and reduced toxicity [19], and overcame the issues of chemotherapy [20]. Of NPs, in the last 25 years, different carbon materials have been studied for different applications [21] in the biomedical field, including drug delivery [22]. Carbon-based materials comprise several allotropic species of carbon. Currently, the most widely used carbon-based nanobiomaterials are carbon nanoparticles, in various forms: carbon nanotubes (CNTs), fullerenes, nanodiamonds, graphene, carbon nano-onions [23]. The best-known carbon-based nanomaterials used in biomedical applications include CNTs and fullerenes. These structures are hydrophobic, therefore different surface modification methods have been used to improve their solubilization [13] in water for biomedical applications [24]. CNTs are cylindrical graphene structures with unique physical and chemical properties, such as high mechanical strength, optical properties, and electrical and thermal conductivity. Moreover, the small size, high surface area-to-volume ratio, high drug-loading capacity and stability, biocompatibility, ease of functionalization, and the ability to penetrate cells and release the drug at a target site without being metabolized have attracted considerable interest for CNTs in the drug delivery field [25,26]. The biomolecules can be covalently grafted or non-covalently adsorbed on the nanotube surface. In addition, the inner core of CNTs can be exploited to encapsulate drugs or NPs [27,28]. Moreover, compared with other types of nanosized drug delivery systems, CNTs present a higher drug-loading capacity due to the high surface area and possibility of conjugating the drug through encapsulation and surface attachment. CNTs enable the engineering of surface modifications for a variety of therapeutic molecules either by specific adsorption or covalent bioconjugation. Another important advantage of CNTs is the interaction with plasmatic membranes and the specific cell internalization mechanism. CNTs could pass the cellular membrane through a “needle-like” cell penetration, in an endocytosis-independent pathway, which provides the advantage of a direct cytoplasmic transport of therapeutic agents [25].

Among CNTs, single-walled carbon nanotubes (SWCNTs) are of considerable interest due to the improvement in the efficiency of drug delivery at the tumor site that they allow. Compared to other carbon nanostructures or metal nanoparticles, SWCNTs present important advantages. Firstly, SWCNTs allow the loading of a large amount of drug, have structural flexibility and intrinsic stability, and are characterized by an improved circulation time and bioavailability. Secondly, SWCNTs are able to transport low-molecular weight biological molecules with a higher cellular penetration rate and without triggering an immune response [29]. On top of that, SWCNTs exhibit IR fluorescence [30]. The IR spectrum between 900 and 1300 nm is an important optical window for biomedical applications due to its lower optical absorption (greater depth of light penetration) and small autofluorescent background, CNTs also displaying good photostability [31]. 

In this context, the current study aimed to evaluate the anti-tumoral efficacy of a SWCNT conjugate, based on double functionalization with carboxyl groups and the anti-tumor drug cisplatin (SWCNT-COOH-CDDP), in the modulation of the PI3K/Akt/mTOR signaling pathway in MDA-MB-231 triple-negative breast cancer cells. SWCNTs were covalently functionalized with carboxyl groups, conjugated with CDDP, and characterized using FTIR, SEM, TEM, and EDS techniques. Biological effects were evaluated after the exposure of MDA-MB-231 cells to nanoconjugates for 24 and 48 h. In vitro results revealed that SWCNT-COOH-CDDP nanoconjugates induced the inhibition of the PI3K/Akt/mTOR pathway, Akt degradation, and inhibition of the cell migration potential of TNBC cells. 

## 2. Materials and Methods

### 2.1. Nanoparticle Functionalization and Characterization

#### 2.1.1. SWCNT Functionalization

SWCNTs were purchased from Sigma-Aldrich (St Louis, MO, USA) having more than 90% carbon basis and D × L 10–15 nm × 0.1–10 μm, produced by catalytic chemical vapor deposition (CCVD). Functionalization of SWCNTs can reduce their toxicity and increase hydrophilicity. The covalent functionalization of SWCNTs with carboxyl groups was performed by chemical oxidation of SWCNTs with a mixture of sulfuric acid and nitric acid (3: 1 molar ratio). After the covalent functionalization of SWCNTs with -COOH groups, CDDP (Sigma-Aldrich, St Louis, MO, USA, >99%) was added to obtain a double functionalization of the SWCNTs.

The sample obtained after functionalization with carboxyl groups, SWCNT-COOH, was further used to obtain functionalization with CDDP. An amount of 100 mg of CDDP was dispersed in 50 mL dimethylformamide (DMF, Fluka Chemie GmbH, Buchs, Switzerland). For the functionalization with CDDP, 0.5 mg SWCNTs were dispersed in 5 mL of CDDP + DMF solution. The resulting mixture was ultrasonicated for 48 h at 500 °C. The resulting mixture was passed through a filter with a pore diameter of 0.2 µm. The obtained filtrate was then washed with chloroform, followed by washing with deionized water to remove any impurities [32]. The obtained sample was then dried at 80 °C in an electric oven and the SWCNT-COOH-CDDP sample was further characterized.

#### 2.1.2. Characterization of the SWCNT-COOH-CDDP Conjugate

Fourier transform infrared (FTIR) spectra of functionalized SWCNTs were registered with a PerkinElmer Spectrum 100 spectrometer (PerkinElmer, Shelton, DC, USA) in a 400 ÷ 4500 cm^−1^ range with 4 cm^−1^ resolution and 32 scans. 

The morphology of SWCNT-COOH and SWCNT-COOH-CDDP was investigated by transmission electron microscopy (TEM) (Philips Electronics, Eindhoven, The Netherlands) analysis with a Philips EM-410 microscope, using 60 kV acceleration. In addition, SEM–EDS analyses were performed using an instrument from FEI, the Quanta FEG650 (FEI, Hillsboro, OR, USA), equipped with a modern energy dispersive spectroscopy (EDS) module. For analysis, a 10 kV voltage was used. Due to the lack of conductivity of the samples, water vapors were inserted in the SEM chamber.

For inductively coupled plasma mass spectrometry (ICP-MS) analysis, a PerkinElmer Elan DRC-e instrument (PerkinElmer, Shelton, DC, USA) was used. The detection limit of the spectrometer is 0.001 μg/g. To study the samples, acid digestion was carried out in a precisely determined volume of 65% HNO_3_ (Merck, Darmstadt, Germany).

According to the literature [33], the encapsulation efficiency (EE) of platinum-based antitumor drugs into carbon nanotubes is to be evaluated using Equation (1):

Encapsulation efficiency% = (M_total drug_ − M_free drug_)/(M_total drug_) × 100 (1)

The EE was calculated using the data obtained by ICP-MS. The initial concentration of Pt ions present in the CDDP solution was recorded before the addition of SWCNTs. After encapsulation, the nanotubes were removed from solution and another determination of the solution was performed in order to investigate the amount of remaining platinum. The amount of platinum represents a fixed percentage in the CDDP molecule. These data were used to calculate the amount of drug encapsulated in SWCNTs.

### 2.2. In Vitro Bioassays

#### 2.2.1. Cell Line Culture 

The human triple-negative breast cancer cell line, MDA-MB-231, derived from an adenocarcinoma of the mammary gland, was purchased from American Type Culture Collection (ATCC, HTB-26™, Manassas, VA, USA). The cell line was grown in monolayer cultures, in 75 cm^2^ culture flasks and Dulbecco’s Modified Eagle Medium (DMEM, 31600-083, Gibco, UK) supplemented with 3.5 g/L glucose, 1.5 g/L NaHCO_3_, 1% penicillin/streptomycin/amphotericin B solution (A5955, Sigma-Aldrich, St. Louis, MO, USA), and 10% fetal bovine serum (10270-106, origin South America, Gibco, Life Technologies, Carlsbad, CA, USA). 

The cell cultures were maintained in a humidified atmosphere (95% air, 5% CO_2_) at a temperature of 37 °C. At 80–90% confluency, the cells were detached from the flask’s surface with a prewarmed 0.25% trypsin–0.53 mM EDTA solution. The culture medium was renewed every two days. 

#### 2.2.2. Culture Cell Treatment 

To evaluate the biological effects induced by the nanoconjugates and the free components, firstly, the MDA-MB-231 cells were seeded in culture plates or flasks and incubated for 24 h to allow their adherence. The cells were treated with various doses of free CDDP (0.00632, 0.0316, 0.0632, 0.158, 0.316, 0.632, 1.26 μg/mL), SWCNT-COOH (0.01, 0.05, 0.1, 0.25, 0.5, 1, 2 μg/mL), and SWCNT-COOH-CDDP (0.01 + 0.00632, 0.05 + 0.0316, 0.1 + 0.0632, 0.25 + 0.158, 0.5 + 0.316, 1 + 0.632, 2 + 1.26 μg/mL) diluted in complete culture medium. Untreated cells were used as control. The in vitro experiments were performed after 24 and 48 h of treatment. 

#### 2.2.3. MTT Cell Viability Test

Cell viability was evaluated using the MTT assay. For this, adenocarcinoma cells were seeded in 24-well plates at a density of 4 × 10^4^ cells/well/mL and incubated at 37 °C, for their adherence. The next day, the cells were exposed to CDDP (0.00632–1.26 μg/mL), SWCNT-COOH (0.01–2 μg/mL) and SWCNT-COOH-CDDP (0.01 + 0.00632–2 + 1.26 μg/mL) and incubated at 37 °C. At the experimental time intervals, the culture medium was removed and the cells were incubated with 300 μL of a 1 mg/mL MTT (M5655, Sigma-Aldrich, St. Louis, MO, USA) solution, at 37 °C, for 2 h. Then, the MTT solution was discarded and the formazan crystals formed as a result of the mitochondrial activity of MDA-MB-231 cells were solubilized with an isopropanol solution. The optical density of samples was measured using a microplate reader (FlexStation 3) at a wavelength of 595 nm. The results were expressed as a percentage of the control (100% viability). 

#### 2.2.4. Live/Dead Assay

To perform the live/dead assay, breast cancer cells were seeded in 24-well plates at a density of 4 × 10^4^ cells/well/mL and incubated at 37 °C, in an atmosphere with 5% CO_2_. After their adherence, the cells were treated with 0.05–1 μg/mL SWCNT-COOH, 0.0316–0.632 μg/mL CDDP and the corresponding doses of nanoconjugate and then incubated at 37 °C. After 24 and 48 h of treatment, the culture medium was removed, and the cells were treated with a serum-free solution containing 2 µM calcein AM and 4 µM ethidium homodimer. After 20 min of incubation at 37 °C, the cells were visualized with an Olympus IX73 microscope (Olympus, Tokyo, Japan) using FITC and TRITC filters. 

#### 2.2.5. Protein Extraction

For the protein extraction protocol, MDA-MB-231 cells were seeded in 75 cm^2^ culture flasks at a density of 10^6^ cells/flask and treated with two different doses of CDDP (0.316 and 0.632 μg/mL), SWCNT-COOH (0.5 and 1 μg/mL), and SWCNT-COOH-CDDP (0.5 + 0.316 and 1 + 0.632 μg/mL). After 24 and 48 h of treatment, the monolayer cultures were enzymatically detached from the culture flasks, the cell suspensions were centrifuged for 5 min, at 1500 rpm, and the cellular pellets were washed and resuspended in phosphate buffer saline (PBS).

Cell lysis was produced on ice, through ultrasonication (three times, 30 s), using a UP50H ultrasonicator (80% amplitude, 1 cycle, Hielscher Ultrasound Technology, Teltow, Germany). Then, the lysates were centrifugated (10 min, at 3000 rpm, 4 °C) and the resultant supernatants were collected and used for protein expression analysis. The protein concentration of the samples was estimated using Bradford reagent and a standard curve of 0–1.5 mg/mL bovine serum albumin (BSA). 

#### 2.2.6. Western Blot Analyses 

The expressions of tumor suppressor protein p53 and the main proteins from the PI3K/Akt/mTOR signaling pathway (PI3K, Akt, p-Akt1, PTEN) were analyzed using the Western blot method. Before electrophoresis, the samples were diluted with PBS depending on protein concentration and prepared by chemical and thermic denaturation. For this, the lysates were mixed with a loading buffer containing sodium dodecyl sulfate (SDS) and β-mercaptoethanol and then incubated on a thermoblock at 95 °C, for 5 min. Then, equal protein amounts (40 μg) were loaded and electrophoresed on 8% and 10% polyacrylamide gels in a TRIS–glycine–SDS buffer, at a voltage of 90 V. Then, the proteins were transferred onto a polyvinylidene fluoride (PVDF) membrane using a BioRad transfer system and a TRIS–glycine–methanol buffer. 

Afterwards, the protein bands were revealed using Western Breeze Chromogenic Anti-Mouse and Anti-Rabbit kits (WB7103, WB7105, Invitrogen, Carlsbad, CA, USA). The membranes were blocked, for 30 min, at room temperature, then blotted overnight with monoclonal anti-PI3K (sc-374534, Santa Cruz, Dallas, TX, USA), anti-Akt (sc-81434, Santa Cruz, Dallas, TX, USA), anti-p-Akt (sc-514032, Santa Cruz, Dallas, TX, USA), anti-PTEN (sc-7974, Santa Cruz, Dallas, TX, USA), anti-β-actin (A1978, Sigma-Aldrich, St. Louis, MO, USA) and polyclonal anti-p53 (sc-6243, Santa Cruz, Dallas, TX, USA) primary antibody solutions. Following the washes and incubation (30 min, at room temperature) with the alkaline phosphatase-conjugated secondary anti-mouse and anti-rabbit antibody solutions, the protein signals of interest were detected using a BCIP/NBT chromogenic substrate. The blot membranes were visualized with a ChemiDoc Imaging System (Bio-Rad, Hercules, CA, USA), and the protein bands were analyzed using ImageLab software. β-actin was used as a reference protein to normalize the results. 

#### 2.2.7. Wound Healing Assay 

The wound healing assay was performed to evaluate the migration potential of MDA-MB-231 cells after treatment. 

TNBC cells were seeded in 24-well plates at a density of 4 × 10^4^ cells/well and incubated at 37 °C to allow cell growth. When cells almost reached confluence, the culture medium was removed and a scratch was manually traced in the middle of each of the wells using a 0.5–10 μL pipette tip. Then, the wells were washed with culture medium to remove the detached cells. Immediately after, the cells were treated with 0.316 and 0.632 μg/mL CDDP, 0.5 and 1 μg/mL SWCNT-COOH, and 0.5 + 0.316 and 1 + 0.632 μg/mL SWCNT-COOH-CDDP. After 0 (T0), 24, and 48 h of treatment, the scratch was monitored by means of optical microscopy using an Olympus IX73 microscope (Olympus, Tokyo, Japan). Image acquirement was performed using the CellSens Dimension software (v1.11, Olympus). The wound closure was measured using ImageJ software (v1.52a, National Institutes of Health, Bethesda, MD, USA) [34] and the results were calculated relative to the T0 moment of the experiment. For each experimental condition, 12 images were analyzed. 

#### 2.2.8. Statistical Analysis 

The results were calculated as an average of three replicates and expressed relative to the control (untreated cells) ± standard deviation. The results were statistically analyzed, comparing treated cells and the control, using the two-way ANOVA method performed with GraphPad Prism (Version 8, GraphPad Software, La Jolla, CA, USA) and Tukey’s multiple comparisons test. The values *p* < 0.05 (*), *p* < 0.01 (**), and *p* < 0.001 (***) were considered significant. 

## 3. Results

### 3.1. FTIR Measurements

**Figure 1:**
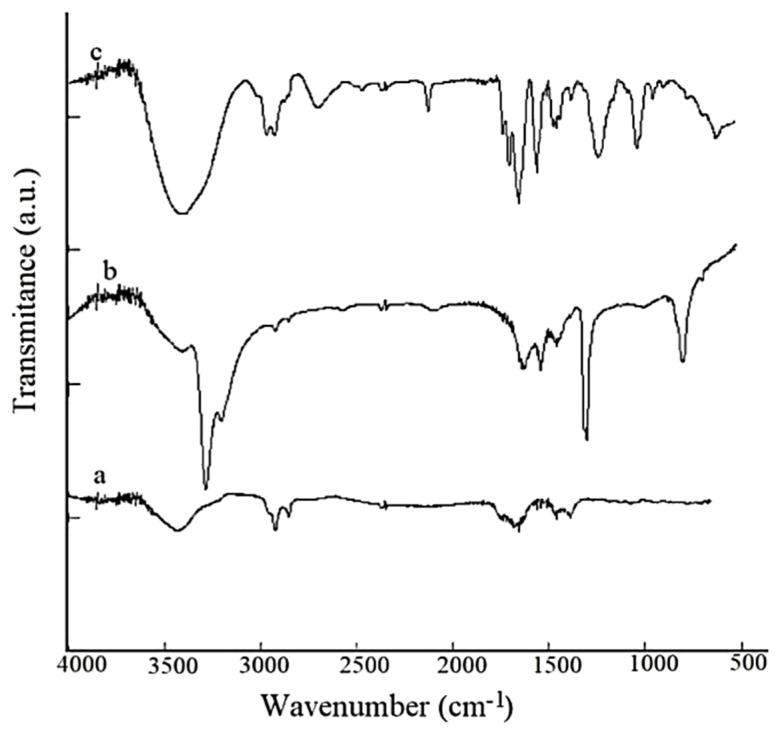
FTIR spectra for (a) SWCNT -COOH; (b) CDDP; (c) SWCNT-COOH-CDDP.

The functionalization with CDDP of the carboxylated SWCNTs is suggested by the records and the comparison of SWCNT-COOH and CDDP spectra (a and b). Fourier transform infrared spectroscopy was used to confirm the covalent binding of carboxyl functional groups on the surface of SWCNTs. The FTIR spectra obtained are shown below, in Figure 1.

FTIR spectroscopy highlighted the presence of functional groups on the surface of SWCNT-COOH and SWCNT-COOH-CDDP samples. The functional groups related to -COOH functionalization appear in a range between 1000 and 2000 cm^−1^. The absorption band at 1634 cm^−1^ is attributed to the C=C bond characteristic of the hexagonal network in SWCNTs. The vibration mode for C–O and C=O, O–H bonds appears in the wavelength range between 1700–1500 cm^−1^. For SWCNT-COOH-CDDP, there is a shift in the spectra to higher frequencies (cm^−1^) compared to the spectra of CDDP, a fact that suggests the functionalization of COOH with –Pt, –COOPt groups. Due to oxidation with HNO_3_, the peak that appears near 1350 cm^−1^ corresponds to Pt-N-H in CDDP spectra and changes at a lower frequency in SWCNT-COOH-CDDP spectra. In CDDP spectra, the peak that appears at 700 cm^−1^ can be attributed to Pt–N. This peak is shifted to a higher frequency, at about 600 cm^−1^, which can be associated with Pt–O in SWCNT-COOH-CDDP.

### 3.2. TEM Measurements

**Figure 2:**
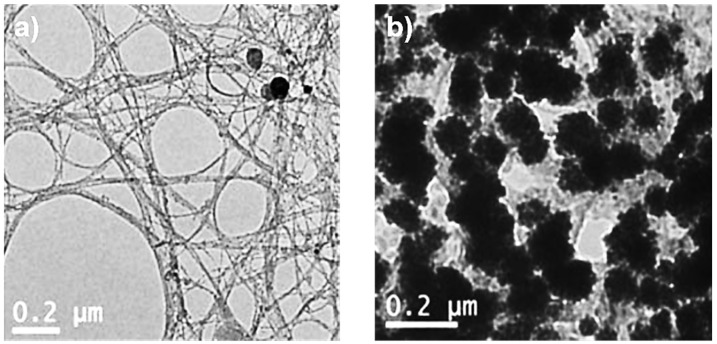
TEM morphologies for: (**a**) SWCNT-COOH; (**b**) SWCNT-COOH-CDDP.

CNT morphologies were investigated using TEM analysis. The surfaces of SWCNT-COOH and SWCNT-COOH-CDDP can be seen in Figure 2.

The carboxylate nanotube morphology reveals similar SWCNTs morphologies, with the tubes bounded, with some catalyst and amorphous carbon particles, and forming a net. 

After functionalization with the therapeutic agent, the morphology significantly changed. In the case of SWCNT-COOH-CDDP (Figure 2b), CDDP can be observed as grains that are 10–20 nm wide and 10–20 nm in length. The SWCNT net is partially or totally covered with CDDP, this morphology playing an important role, as a template for anticancer drug molecules, such as CDDP.

### 3.3. SEM–EDS Measurements

**Figure 3:**
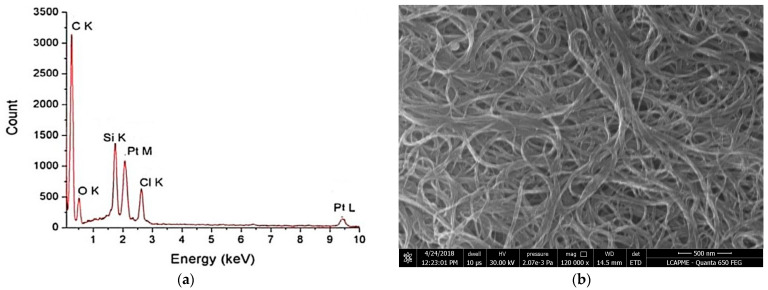
(**a**) EDS and (**b**) SEM morphologies for SWCNT-COOH-CDDP.

Figure 3 shows the EDS spectra and the SEM morphology for the doubled functionalized SWCNTs. 

In Figure 3b, the platinum appears as lighter spots in the images because of the higher atomic number (Z) compared with carbon. The Si peak does not represent the SWCNT sample but an artifact signal from the glass plate on which the CNTs were deposited for the analysis. The EDS (Figure 3a) evidences the carbon from pristine SWCNTs, as well as the platinum and chloride (from CDDP). The EDS also determined the Pt:Cl molar ratio (of 1:1).

### 3.4. ICP-MS Measurements

These studies were conducted in PBS using a pH of 5.5 (typical for the cancer environment). A volume of 1 mL SWCNT-CDDP sample was immersed in 20 mL PBS for different period of times. Then, 2 mL of the PBS-diluted SWCNT-COOH-CDDP sample was collected from the release medium at regular intervals. The platinum content was determined using ICP-MS to evaluate CDDP released from SWCNT-COOH-CDDP. 

**Figure 4:**
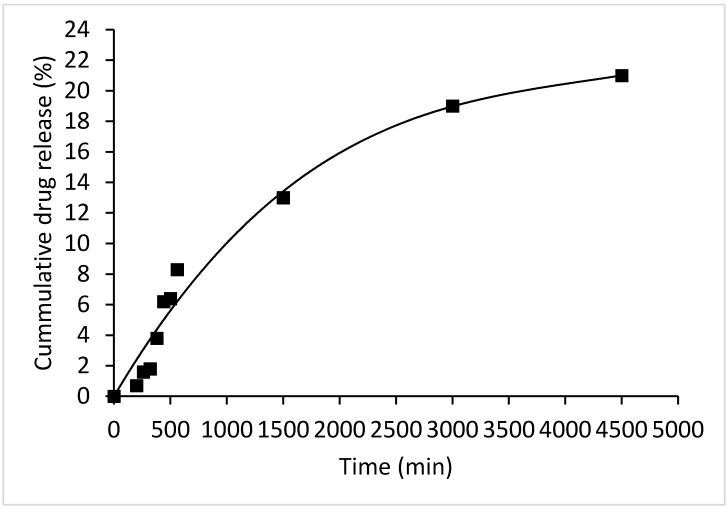
Release profile of CDDP from SWCNT-COOH-CDDP at pH = 5.5.

For the ICP-MS analysis, the samples containing Pt ions (a volume of 2 mL) were digested in 200 mL concentrated nitric acid p.a. from Sigma-Aldrich (St. Louis, MO, USA). After digestion in nitric acid, the samples were diluted 100 times with ultrapure water, and liquid fractions were analyzed [13]. Platinum (20 mg/L) was used as an internal standard for the calibration curve and to analyze solutions in ICP-MS. ICP-MS was performed to calculate platinum’s encapsulation efficiency. Measurements for CDDP loading were performed for SWCNT-COOH-CDDP samples following the protocol. From ICP-MS measurements, evidence was obtained for the concentration of Pt ions in the domain of µg/mL. The ICP-MS results indicate that the loaded CDDP was 21 ± 0.4%. The release profile of CDDP from SWCNT-COOH up to 72 h (4500 min) is shown in Figure 4, indicating a rapid release in the first 24 h.

### 3.5. SWCNT-COOH-CDDP Decreases Cell Viability and Induces Cell Death in the MDA-MB-231 Culture

**Figure 5:**
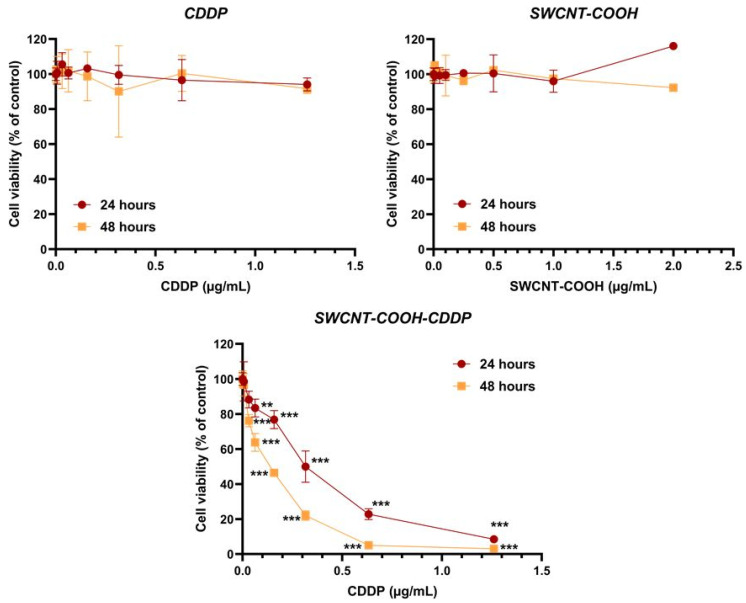
MDA-MB-231 cell viability after the treatment with CDDP (0.00632–1.26 μg/mL), SWCNT-COOH (0.01–2 μg/mL), and SWCNT-COOH-CDDP (0.01 + 0.00632–2 + 1.26 μg/mL) for 24 and 48 h. The results are expressed as percentages relative to control (100%) ± standard deviation (SD) and were considered statistically significant when *p* < 0.01 (**), *p* < 0.001 (***) (sample vs. control).

MDA-MB-231 cell culture viability was estimated after the treatment with the nanoconjugates and free components for 24 and 48 h. The results presented in Figure 5 indicate that nanoconjugates induced a decrease in cell viability in a time and dose-dependent manner, with the highest decrease after 48 h of treatment with a dose of 2 + 1. 26 μg/mL SWCNT-COOH-CDDP, highlighting a pronounced cytotoxic effect. The decrease of cell viability until 50% was registered after 24 and 48 h of treatment with a dose of 0.5 + 0.316 μg/mL and 0.25 + 0.158 μg/mL SWCNT-COOH-CDDP, respectively. Simultaneously, a free CDDP sample did not induce significant modifications of cell viability relative to untreated cells. The same tendency was observed after the treatment with the SWCNT-COOH sample, cell viability remaining almost at control level, illustrating a good biocompatibility of this sample. 

**Figure 6:**
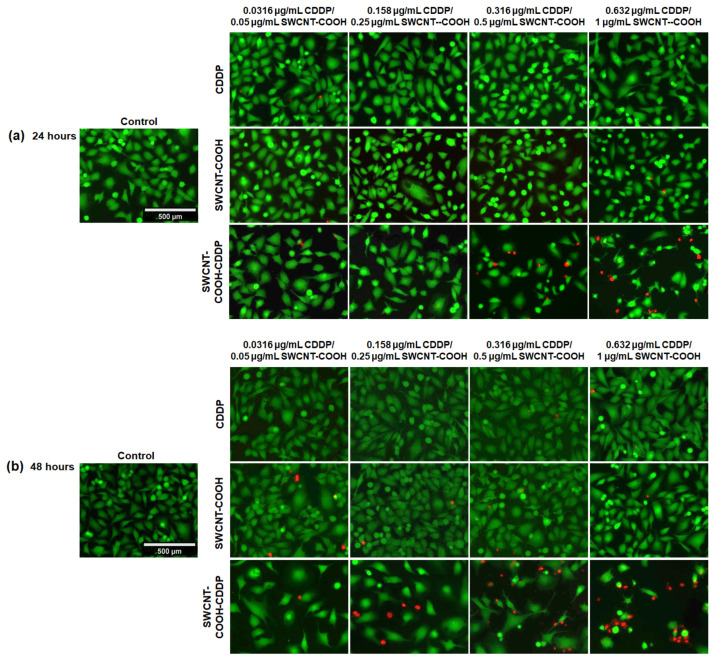
Fluorescence labeling of MDA-MB-231 cells with 2 µM calcein AM and 4 µM ethidium homodimer after (**a**) 24 and (**b**) 48 h of treatment with CDDP (0.0316–0.632 μg/mL), SWCNT-COOH (0.05–1 μg/mL), and SWCNT-COOH-CDDP (0.05 + 0.0316–1 + 0.632 μg/mL). Green fluorescence indicates live cells and red fluorescence indicates dead cells. The scale bar = 500 μm and is the same for all images.

We explored the presence of dead cells in the MDA-MB-231 cultures exposed to treatment. The fluorescence images presented in Figure 6 illustrate the presence of dead cells, indicated by the red fluorescence, after the treatment with the highest doses of SWCNT-COOH-CDDP (0.5 μg/mL SWCNT-COOH + 0.316 μg/mL CDDP and 1 μg/mL SWCNT-COOH + 0.632 μg/mL CDDP) for 24 h. Moreover, an increase in dead cell numbers dependent on dose was registered also at the interval of 48 h, starting with a lower dose of SWCNT-COOH-CDDP corresponding to 0.25 μg/mL SWCNT-COOH + 0.158 μg/mL CDDP. The presence of dead cells was accompanied by a reduction of viable cells, as indicated by green fluorescence, suggesting the potential of SWCNT-COOH-CDDP to induce cell death and inhibit the growth rate of MDA-MB-231 cultures. In accordance with the results of the MTT test, the live/dead assay revealed that free CDDP and SWCNT-COOH did not induce significant modifications of breast cancer cells, the labels being almost similar to the control’s. 

After the evaluation of cell viability and death, two doses of nanoconjugates—SWCNT-COOH-CDDP: 0.5 + 0.316 μg/mL (low dose) and 1 + 0.632 μg/mL (high dose)—and the corresponding doses of free CDDP and SWCNT-COOH were chosen for further experiments. 

### 3.6. SWCNT-COOH-CDDP Induces the Inhibition of PI3K, p-Akt, and Akt Proteins and Elevation of PTEN Expression in TNBC Cells 

The effects of SWCNT-COOH-CDDP on proliferation and migration signaling were studied by analyzing the expression of p53, PI3K, p-Akt1, Akt, and PTEN proteins. The results revealed a slight decrease of p53 protein expression after 24 h of treatment with the high dose of the nanoconjugate. In contrast, at 48 h, the expression of p53 increased after the treatment with 0.5 + 0.316 μg/mL SWCNT-COOH-CDDP, which can indicate the activation of the translation of this protein in the presence of a low dose of nanoconjugate. In addition, a slight increase of p53 expression was obtained in the presence of 0.632 μg/mL free CDDP after both time intervals. 

We further analyzed the expression of PI3K, which was inhibited in the presence of the nanoconjugate after 24 h of treatment with the high dose and after 48 h of incubation with the two tested doses. In correlation with the inhibition of PI3K, the expression of p-Akt1 significantly decreased in the presence of SWCNT-COOH-CDDP after 24 h of incubation with a dose of 1 + 0.632 μg/mL and in a dose-dependent manner after 48 h, suggesting the inhibition of PI3K/Akt signaling in these experimental conditions. 

**Figure 7:**
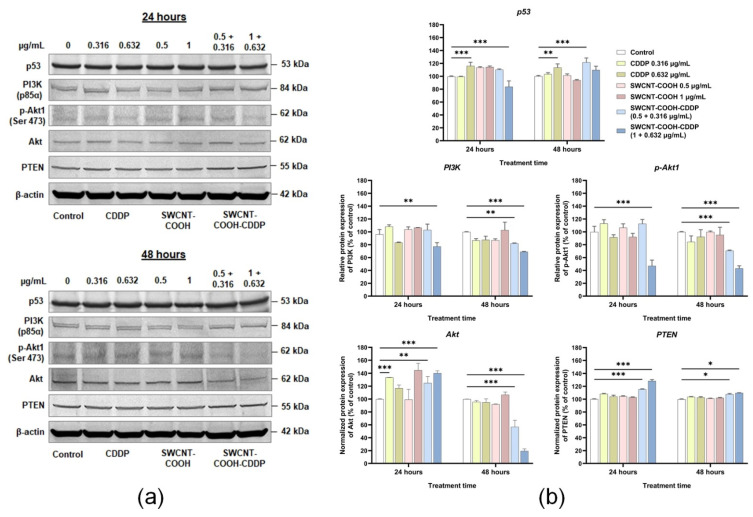
(**a**) Western blot images representing the protein expressions of p53, PI3K, p-Akt, Akt, PTEN, and β-actin after 24 and 48 h of treatment with CDDP (0.316, 0.632 μg/mL), SWCNT-COOH (0.5, 1 μg/mL), and SWCNT-COOH-CDDP (0.5 + 0.316, 1 + 0.632 μg/mL). (**b**) Quantification of blot images. Data were normalized to β-actin and expressed as percentages related to control (100%) ± standard deviation (SD). The results were considered statistically significant when *p* < 0.05 (*), *p* < 0.01 (**), *p* < 0.001 (***) (sample vs. control).

Simultaneously with PI3K and p-Akt1 inhibition, the expression of PTEN protein increased in the presence of the two doses of the nanoconjugate, compared to untreated cells, after 24 and 48 h of treatment, supporting the above conclusion regarding PI3K signaling inhibition. Moreover, Akt protein expression presented an increase after the incubation for 24 h with the SWCNT-COOH-CDDP sample, followed by inhibition at 48 h, observed in the case of both doses, suggesting a possible degradation of this protein after the exposure to nanoconjugates. An elevation of Akt expression was also registered after 24 h of treatment with 0.316 μg/mL CDDP. Regarding the SWCNT-COOH sample, no significant changes were obtained in any of the experimental conditions (Figure 7).

### 3.7. SWCNT-COOH-CDDP Nanoconjugates Inhibit the Migration Potential of MDA-MB-231 Cells

**Figure 8:**
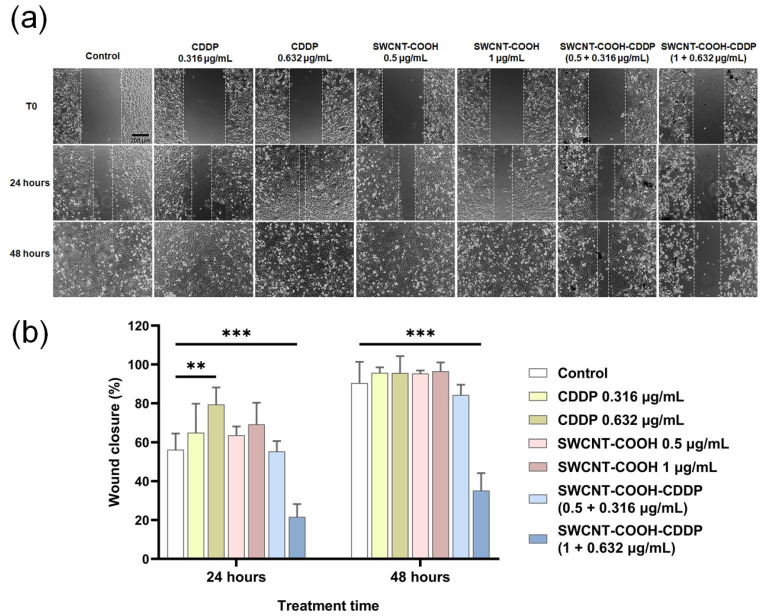
(**a**) Wound healing assay illustrating changes in the migration rates of MDA-MB-231 cells after the treatment with 0.316, 0.632 μg/mL CDDP, 0.5, 1 μg/mL SWCNT-COOH, and 0.5 + 0.316 μg/mL, 1 + 0.632 μg/mL SWCNT-COOH-CDDP for 0 (T0), 24, and 48 h. Scale: 200 μm. (**b**) The graph is the corresponding quantification of wound healing images presented. Columns represent mean values ± standard deviation (SD) relative to T0. The results were considered statistically significant when *p* < 0.01 (**), *p* < 0.001 (***) (sample vs. control).

In order to evaluate the motility of MDA-MB-231 cells after 24 and 48 h of treatment, the wound healing assay was performed. In the presence of SWCNT-COOH-CDDP nanoconjugates, the migration efficiency of the cells treated with 1 + 0.632 μg/mL SWCNT-COOH-CDDP was inhibited in a time-dependent manner (by 34.58% and 55.28% relative to the T0 moment, after 24 and 48 h of treatment, respectively). These results could highlight the efficiency of SWCNT-COOH-CDDP in reducing the migration rate of MDA-MB-231 cells. However, a dose of 0.5 + 0.316 μg/mL SWCNT-COOH-CDDP did not affect the migration of TNBC cells. The free components did not induce significant modifications, except for the high dose of free CDDP, which induced an increase of 23.21% in migration potential after 24 h of treatment (Figure 8), illustrating a migration-promoting effect of free CDDP. 

## 4. Discussion

The PI3K/Akt signaling pathway has a fundamental role in the progress and development of tumors and resistance to chemotherapies. In TNBC, the signaling through PI3K and Akt represents one of the most frequent dysregulated pathways and is associated with the oncogenic phenotype and uncontrolled overgrowth of tumors [8]. Consequently, targeting the major nodes of this signaling pathway is being used as a strategy to increase the efficiency of treatments. In this context, the current study aimed to synthesize and characterize a nanoconjugate based on SWCNT-COOH and CDDP and to investigate its in vitro anti-tumor potential by analyzing the efficiency of its inhibition of the PI3K/Akt signaling pathway and the motility of TNBC cells. 

Firstly, SWCNTs were chemically functionalized with -COOH groups and highlighted by FTIR measurements. The chemical functionalization was performed to support the attachment of the chemotherapeutic agent CDDP to the carbon nanotube structures. Functionalized SWCNTs were visualized using TEM and SEM microscopy, by means of which the surface of the sample was clearly observed. The presence of Pt ions was highlighted by EDS analysis and the encapsulation efficiency of the drug was studied by ICP-MS analysis.

CDDP cytotoxicity is associated with DNA strand interaction and adduct formation, which lead to cell cycle arrest and cell death through apoptosis [35]. In our study, the evaluation of cytotoxicity through cell viability analysis revealed the high biocompatibility of SWCNT-COOH and a pronounced cytotoxic effect of the nanoconjugate compared with the free drug. The cytotoxicity of the nanoconjugate was dependent on the concentration applied on cells and on exposure time. A dose-dependent reduction of MDA-MB-231 cell viability was reported, also, for higher concentrations of CDDP: 25–100 µM (corresponding to 7.5–30 μg/mL) [36]. However, Wawruszak and collaborators (2015) [37] found that a dose of 4 μg/mL CDDP and an interval of 96 h of treatment were necessary to reduce the cell population by half in MDA-MB-231 cultures. Thus, the decrease of half-maximal inhibitory concentration (IC50) of CDDP observed in this case might be explained by conjugation with SWCNTs.

The biocompatibility and high efficiency of CNTs in drug intracellular transport and the improvement of chemotherapeutic action were reported in other papers. For example, Guven and collaborators (2017) [38] reported the superior efficiency of CDDP loaded onto ultra-short single-walled carbon nanotube capsules, compared with free CDDP, by demonstrating a greater efficiency in suppressing tumor growth and a higher accumulation in tumors.

The biocompatibility of CNTs is an important and controversial topic discussed in various papers. Some studies reported the potentially toxic effects of CNTs, while others demonstrated a good biocompatibility and insignificant cellular responses after their incubation with various cell types or administration to animal models. Over the year, CNTs have been associated with different mechanisms of toxicity, such as oxidative stress, inflammatory responses, malignant transformation, DNA damage and mutation, formation of granulomas, induction of apoptosis or necrosis, and interstitial fibrosis. However, the occurrence and degree of toxicity depend on the physical and chemical characteristics of CNTs: surface charge and modifications, size, shape, agglomeration tendency, number of layers, or the presence of impurities [39]. To mitigate toxicity, CNTs were functionalized with amino and carboxyl groups, polyethylene glycol, or phospholipid–polyethylene glycol [40]. Previous studies have shown that functionalized and individualized SWCNTs (SWCNTs that present a reduced agglomeration tendency) could be non-toxic and well-tolerated in vivo [41].

The results obtained after the cell viability evaluation were confirmed by the fluorescence labeling of live and dead cells. The presence of dead cells after the treatment of MDA-MB-231 with the nanoconjugates is a proof of cell death induction in MDA-MB-231 cells, most probably through apoptotic or necrotic pathways. 

Furthermore, we explored the modifications in protein expression of major markers involved in the PI3K/Akt signaling pathway correlated with tumor growth, proliferation, and survival. PI3K are plasma membrane-associated lipid kinases, which according to their structural characteristics and substrate specificity are grouped into three main classes: I, II, and III [7,42]. Class I PI3K is divided into IA and IB PI3K. Class IA PI3K molecules, which have an important involvement in human cancer, are heterodimers consisting of a p110 catalytic subunit and p85α, p85β, and p55γ (collectively called p85) regulatory subunits [42]. Hence, we quantified the expression of the p85α regulatory subunit. The expression of the p85α regulatory subunit of PI3K was significantly inhibited after the treatment of breast cancer cells with the high dose of nanoconjugates (1 μg/mL SWCNT-COOH + 0.632 μg/mL CDDP) for 24 h and after 48 h with both doses. 

In PI3K signaling, the activation of receptor tyrosine kinase (RTK) activates PI3K, inducing the conversion of PIP2 molecules to PIP3 ones, which represent new anchoring sites for downstream signaling proteins, 3-phosphoinositide-dependent kinase 1 (PDK-1) and serine–threonine protein kinase Akt, and their membrane association. At the membrane, PDK-1 phosphorylates the Akt protein, and its activation stimulates cell growth and proliferation through the PDK-1/Akt-mediated pathway [43]. After PI3K protein expression quantification, we explored the expression of p-Akt1 and, as expected, an inhibition of this protein was also found in the same experimental conditions. The antibody (C-11) used against p-Akt1 is recommended for the detection of Ser473 phosphorylated Akt1. The phosphorylation of Ser473 induces the full activation of the Akt protein after its phosphorylation at the Thr308 residue [44,45]. Considering this, the decreased expression of p-Akt1 after the exposure of MDA-MB-231 cells to treatment might be a consequence of PI3K inhibition. Moreover, the downregulation of both PI3K and p-Akt1 proteins could lead to the first hypothesis of our study, which suggests that the death of MDA-MB-231 cells is caused by the inhibition of PI3K/Akt signaling as a result of SWCNT-COOH-CDDP nanoconjugate exposure. Our results are supported by previous data that indicate a correlation between PI3K/Akt pathway inhibition and the overcoming and reversal of CDDP resistance in TNBC [46] and lung [47] cancer cell lines. Moreover, the inhibition of the PI3K/Akt pathway using specific inhibitors is studied as a therapeutic strategy in solid tumor treatment [48]. The phosphorylation of Akt proteins leads to the phosphorylation of other proteins, this mechanism being associated with cell proliferation and survival [45]. Thus, the decrease in cell viability registered in the presence of SWCNT-COOH-CDDP nanoconjugates might be a consequence of p-Akt1 downregulation.

In cells, PI3K activity is counterbalanced by the PTEN suppressor protein, which induces the negative regulation of this pathway [49]. In cancer cells, PTEN gene is frequently mutated, thus leading to uncontrolled growth, cancer susceptibility, and progression [50]. Several studies revealed that the systemic elevation of PTEN protein promotes the suppression of the tumor state [51]. Additionally, in TNBC, the poor prognosis is associated with PTEN reduced/PI3K high/m-TOR high expression [49]. Consistent with these studies, we obtained an upregulation of PTEN protein expression after the treatment of MDA-MB-231 cells with the nanoconjugates, a result that could suggest the inhibition of tumor growth by PI3K signaling inhibition. This result correlates with the downregulation of PI3K and p-Akt1 protein expression.

Modulation of PI3K, p-Akt1, and PTEN expression was accompanied by Akt protein upregulation at 24 h, followed by a strong downregulation at 48 h, after the treatment with the CDDP conjugated with SWCNT-COOH. In addition, an elevation of Akt proteins was registered in the presence of a low dose of free CDDP after 24 h. Generally, the overexpression of Akt is associated with tumorigenesis, a malignant phenotype [52], and chemoresistance [53]. Thus, the slight increase of Akt protein levels could suggest a cellular tendency to counteract the effects induced by CDDP treatment. Instead, the inhibition of Akt protein expression after 48 h of incubation with the nanoconjugates might occur possibly as a result of the degradation of the protein in these experimental conditions. The mechanism of Akt protein degradation can be induced by the proteasome pathway and caspase-3-dependent proteolysis [54] or calpains action [55]. Another study conducted by Wei and collaborators (2019) [56] reported the proteolysis of this kinase and inhibition of p-Akt (Ser473) in pancreatic cancer (PANC-1 and MIA PaCa-2) cell lines exposed to epigallocatechin-3-gallate and gemcitabine treatment. In their study, the decrease of Akt activation was correlated with the blockade of protein transcription, translation, and degradation [56]. Moreover, it was reported that the involvement of Akt downregulation in the cell death of metastatic skin carcinoma was induced by CDDP [57]. In our study, the decreased expression of p-Akt1 proteins might occur, also, as a result of Akt protein degradation, which could indicate the inhibition of PI3K/Akt signaling through multiple pathways that involved PI3K downregulation and Akt degradation.

One important marker connected to PI3K/Akt signaling is the p53 protein. In this regard, it is considered that p53 proteins can suppress the phosphorylation of downstream factors of PI3K [58]. Simultaneously, it was demonstrated that inhibition of PI3K signaling using PI3K inhibitors prevents p53 induction [59]. In our study, p53 protein expression registered a slight decrease in the presence of the high dose of SWCNT-COOH-CDDP after 24 h and reached the control level at 48 h. Moreover, the low dose of nanoconjugate induced the elevation of p53 protein expression after 48 h. However, the results obtained in these experimental conditions indicate the inhibition of PI3K signaling in breast cancer cells. We assumed that in the presence of the low dose of nanoconjugate, the inhibition of PI3K occurred through p53 activation, while the high dose induced the activation of a different mechanism in a p53-independent manner. 

Besides cell growth, proliferation, and survival, the PI3K/Akt axis is also involved in cell migration and invasion through Akt activation. The role of this signaling pathway in cell motility was highlighted in ovarian cancer cells [60] and endometrial cells [61], but also in breast cancer cells [62,63]. Here, we found that treatment with SWCNT-COOH-CDDP (1 μg/mL SWCNT-COOH + 0.632 μg/mL CDDP) induced suppression of MDA-MB-231 cell motility after both intervals tested, which might be explained by the inhibition of the PI3K/Akt pathway in these conditions. Interestingly, we observed an increase in cell migration potential after 24 h of treatment with the high dose of free CDDP. This result might indicate a stimulation of cell motility and simultaneously highlights the efficiency of SWCNT-COOH in the delivery of CDDP at the intracellular level. From these data, our second hypothesis resulted, according to which suppression of breast cancer cell migration occurs as a consequence of PI3K signaling inhibition.

In the present study, we proved the high tendency of CDDP delivered into TNBC cells using SWCNT-COOH systems to induce cell death through downregulation of PI3K/Akt signaling in TNBC cells. Our findings are in accordance with similar studies which used platinum NPs conjugated with doxorubicin [64] and gold NPs conjugated with quercetin [65] that proved to have a significant therapeutic efficacy against breast cancer cells through PI3K/Akt signaling downregulation. To our knowledge, the current study is the first that explored the efficiency of CDDP inhibition of the PI3K/Akt signaling pathway using carbon nanotubes as drug delivery systems. Carboxyl-functionalized SWCNT loaded with CDDP could be used as a promising approach to extend the benefits in the treatment of TNBC by developing more specific therapeutic strategies. Moreover, our results can represent a scientific fundament to increase CDDP therapy responsiveness and contribute to understanding the molecular mechanisms triggered in breast cancer cells exposed to this chemotherapeutic agent.

## 5. Conclusions

In this study, we referred to CDDP, a platinum-based drug used in cancer therapy which has been used for conjugation with carboxyl-functionalized SWCNTs. 

The CDDP loading efficiency of carboxyl-functionalized SWCNTs was 21 ± 0.4%. The ICP-MS measurements revealed a rapid release of CDDP from SWCNT-COOH-CDDP in the first 24 h with a maximum reached after 72 h. Our data revealed the following main conclusions: (1) breast cancer cell viability decreased in a time- and dose-dependent manner after treatment with SWCNT-COOH-CDDP compared with free CDDP and SWCNT-COOH, which did not induced significant modifications; (2) the nanoconjugates induced cell death in MDA-MB-231 cultures after 24 and 48 h of treatment, with a dose of 0.5 μg/mL SWCNT-COOH + 0.316 μg/mL CDDP and 0.25 μg/mL SWCNT-COOH + 0.158 μg/mL CDDP, respectively; (3) the nanoconjugates induced the downregulation of PI3K and p-Akt1 protein expression and the upregulation of PTEN expression; (4) the expression of Akt protein decreased after 48 h of treatment with doses of 0.5 μg/mL SWCNT-COOH + 0.316 μg/mL CDDP and 1 μg/mL SWCNT-COOH + 0.632 μg/mL CDDP, indicating possible protein degradation in these experimental conditions; (5) the migration potential of MDA-MB-231 was inhibited in the presence of SWCNT-COOH-CDDP after both time intervals with a dose of 1 μg/mL SWCNT-COOH + 0.632 μg/mL CDDP. 

The results of our study reflect the high potential of CDDP conjugated with SWCNT-COOH in promoting cell death, downregulation of PI3K/Akt signaling, and inhibition of breast cancer cell migration, and could contribute to the development of new strategies for targeted treatment against highly proliferative and metastatic breast cancer.
