# Correction: Badea et al. Carboxyl-Functionalized Carbon Nanotubes Loaded with Cisplatin Promote the Inhibition of PI3K/Akt Pathway and Suppress the Migration of Breast Cancer Cells. *Pharmaceutics* 2022, *14*, 469

**DOI:** 10.3390/pharmaceutics17111376

**Published:** 2025-10-24

**Authors:** Madalina Andreea Badea, Mihaela Balas, Mariana Prodana, Florentina Gina Cojocaru, Daniela Ionita, Anca Dinischiotu

**Affiliations:** 1 Department of Biochemistry and Molecular Biology, Faculty of Biology, University of Bucharest, 91-95 Splaiul Independentei, R-050095 Bucharest, Romania; madalina.andreea.badea@drd.unibuc.ro (M.A.B.); anca.dinischiotu@bio.unibuc.ro (A.D.); 2 Department of General Chemistry, Faculty of Applied Chemistry and Materials Science, Politehnica University of Bucharest, 313 Splaiul Independentei, R-060042 Bucharest, Romania; mariana.prodana@upb.ro (M.P.); daniela.ionita@upb.ro (D.I.); 3 Department of Anatomy, Physiology and Biophysics, Faculty of Biology, University of Bucharest, 91-95 Splaiul Independentei, R-050095 Bucharest, Romania; florentina.cojocaru@unibuc.ro

**Error in Figure**

In the original publication [1], there was a mistake in Figure 3 as published. The reason for this error is that the samples were made at the same time during project PN-III-P2-2.1-PED-2016-0904 (183PED/2017) for both multi-walled and single-walled samples, and there was a mix-up in their names.

The SEM image initially used in the article illustrates multi-walled carbon nanotubes (MWCNTs) and has previously been used in another context, in which they were explicitly analyzed.

Unfortunately, the image was introduced by error in the current work as a result of confusion at the level of graphic selection. We mention that sets of images for single-walled carbon nanotubes (SWCNTs) and MWCNTs samples were acquired in the same SEM session. Further, at conventional resolutions, the morphological differences between SWCNTs and MWCNTs are difficult to notice, which contributed to the confusion. However, in order to ensure a correct structural characterization, the article also includes a TEM analysis, which offers the necessary resolution to confirm the SWCNT-type stack used in the study. Thus, the scientific conclusions remain valid, being supported by the relevant data. For scientific correctness and to avoid any potential ambiguity, we replaced the SEM image in the article with the correct one, corresponding to the SWCNTs characteristics analyzed in this study. The corrected Figure 3 appears below. The authors state that the scientific conclusions are unaffected. This correction was approved by the Academic Editor. The original publication has also been updated.

## Figures and Tables

**Figure 3:**
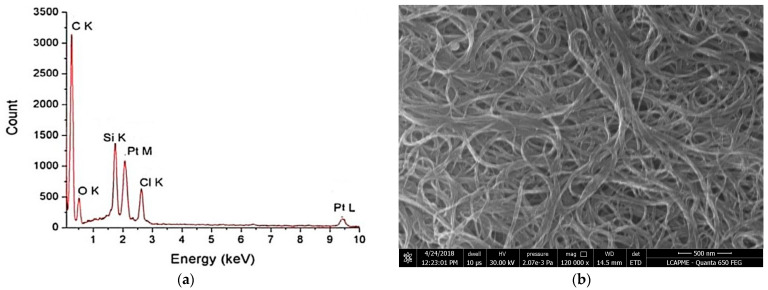
(**a**) EDS and (**b**) SEM morphologies for SWCNT-COOH-CDDP.
